# Improvements in Objective and Subjective Measures of Chronic Cough with Gefapixant: A Pooled Phase 3 Efficacy Analysis of Predefined Subgroups

**DOI:** 10.1007/s00408-022-00553-y

**Published:** 2022-07-27

**Authors:** Jaclyn A. Smith, Surinder S. Birring, Peter V. Dicpinigaitis, Lorcan P. McGarvey, Alyn H. Morice, Ian D. Pavord, Imran Satia, Stuart Green, Beata Iskold, Carmen La Rosa, Qing Li, Allison Martin Nguyen, Jonathan Schelfhout, David Muccino

**Affiliations:** 1 Division of Infection, Immunity & Respiratory Medicine, University of Manchester & Manchester University NHS Trust, 2nd Floor Education & Research Centre, Southmoor Rd, Wythenshawe, Manchester, M23 9LT UK; 2 Centre for Human & Applied Physiological Sciences, School of Basic & Medical Biosciences, Faculty of Life Sciences & Medicine, King’s College London, London, UK; 3 Albert Einstein College of Medicine & Montefiore Medical Center, Bronx, NY USA; 4 Wellcome-Wolfson Institute for Experimental Medicine, School of Medicine, Dentistry & Biomedical Science, Queen’s University Belfast, Belfast, Northern Ireland UK; 5 Hull York Medical School, Cottingham, UK; 6 Oxford NIHR Respiratory BRC, Nuffield Department of Medicine, University of Oxford, Oxford, UK; 7 Department of Medicine, McMaster University, Hamilton, ON Canada; 8 Firestone Institute for Respiratory Health, St Joseph’s Healthcare, Hamilton, ON Canada; 9 Merck & Co., Inc., Rahway, NJ USA

**Keywords:** Antitussives, Cough frequency, Patient-reported outcomes, P2X3-receptor antagonist, Refractory chronic cough, Unexplained chronic cough

## Abstract

**Introduction:**

In phase 3 trials (COUGH-1/COUGH-2), gefapixant 45 mg twice daily significantly reduced 24-h cough frequency vs placebo in refractory or unexplained chronic cough (RCC or UCC).

**Methods:**

Here, the efficacy of gefapixant 45 mg vs placebo was evaluated across COUGH-1/COUGH-2 in predefined subgroups based on sex, region, age, cough duration, cough severity, cough frequency, and diagnosis (RCC, UCC). Awake cough frequency reductions at Week 12 and LCQ response rates (i.e., ≥ 1.3-point improvement) at Week 24 were assessed.

**Results:**

Among 1360 participants analyzed, gefapixant 45 mg resulted in consistent awake cough frequency reductions overall and across predefined subgroups at Week 12. Gefapixant also resulted in improved LCQ scores across subgroups at Week 24; ≥ 70% of participants in each subgroup treated with gefapixant 45 mg had an LCQ response.

**Conclusion:**

These data suggest gefapixant 45 mg provides consistent objective and subjective efficacy across subgroups of individuals with RCC or UCC.

## Introduction

Chronic cough (cough lasting > 8 weeks), a burdensome condition that negatively affects quality of life (QOL) [1], has a prevalence between 4 and 18% [2, 3]. Some patients experience chronic cough associated with certain conditions (e.g., asthma, gastroesophageal reflux disease, upper-airway cough syndrome). However, others experience chronic cough that does not resolve despite extensive investigation and appropriate treatment of comorbid conditions [refractory chronic cough (RCC)] or experience chronic cough that has no identifiable associated conditions despite thorough diagnostic workup [unexplained chronic cough (UCC)] [4, 5]. There is a lack of treatments indicated for RCC or UCC. Off-label treatments may have undesirable side effects [6, 7], and patients may have limited accessibility to nonpharmacologic interventions (e.g., speech therapy) [8]. Treatments that improve both objective cough frequency and subjective cough-specific QOL are needed.

Gefapixant is an oral, peripherally acting P2X3-receptor antagonist under investigation for RCC and UCC [9]. Two phase 3, randomized, placebo-controlled trials of gefapixant showed that relative to placebo, twice-daily gefapixant 45 mg resulted in statistically significant reductions in the primary endpoint, 24-h cough frequency, after 12 (COUGH-1) and 24 (COUGH-2) weeks of treatment. The pooled 24-h cough frequency response was consistent across predefined subgroups at Week 12 [10]. The proportion of participants with a clinically meaningful increase from baseline in the Leicester Cough Questionnaire (LCQ) total score, a key secondary endpoint of interest, was significantly improved after 24 weeks of treatment with gefapixant 45 mg vs placebo in COUGH-2 [10].

This analysis further investigated the efficacy of gefapixant, as assessed via 24-h cough frequency and secondary endpoints of awake cough frequency and the LCQ, across a pooled sample of participants from COUGH-1 and COUGH-2, including in predefined subgroups.

## Methods

### Participants

Participants were enrolled in one of two phase 3, randomized, placebo-controlled, double-blind, parallel-group clinical trials evaluating the P2X3-receptor antagonist gefapixant for treatment of RCC or UCC (COUGH-1, NCT03449134, COUGH-2, NCT03449147). Eligibility criteria have been described, including age of  ≥ 18 years, chronic cough lasting  ≥ 1 year, self-reported score of  ≥ 40 mm on the cough severity visual analog scale (VAS), and RCC or UCC diagnosis per American College of Chest Physicians guidelines [11].

### Study Design

The design and rationale of COUGH-1 and COUGH-2 have been reported [11]. The main study periods were 12 (COUGH-1) and 24 (COUGH-2) weeks. Previous predefined analyses assessed the primary endpoint, 24-h cough frequency, in predefined subgroups including sex, region (North America, Europe, Asia Pacific, other), age (< 65, ≥ 65 years), cough duration (< 10, ≥ 10 years), cough severity VAS (< 60, ≥ 60 mm), 24-h coughs/h (< 20, ≥ 20 coughs/h), and primary diagnosis (RCC, UCC) [10]. The current analysis assessed additional endpoints (awake cough frequency and LCQ) in the same predefined subgroups.

Because cough frequency counts were obtained through Week 12 in COUGH-1 and Week 24 in COUGH-2, pooled awake cough frequency in the current analysis is reported for Week 12 (i.e., the last shared time point across trials for cough frequency measurements). Awake cough frequency was assessed by trained cough analysts. To distinguish awake and sleep periods, Vitalograph analysts defined the starting and ending times for the longest sleep period in each 24-h recording (inclusive of awake periods < 20 min) by considering activity levels, speech, and sounds associated with sleeping (e.g., breathing patterns, snoring). 24-h cough frequency subgroup analyses, but not overall pooled 24-h cough frequency results, were previously reported [10]. For the current analysis, change from baseline in log-transformed cough frequency at Week 12 was evaluated using longitudinal analysis of covariance for the pooled sample (both 24-h and awake cough frequency) and each subgroup (awake cough frequency). Estimated relative reductions from baseline to Week 12 for gefapixant relative to placebo (with 95% CIs) are reported across subgroups. As Week 24 was the time point used for the LCQ as a key secondary endpoint in COUGH-2, data for the LCQ were pooled at Week 24. The proportion of participants with a ≥ 1.3-point increase from baseline to Week 24 on the LCQ total score was evaluated using a logistic regression model for the pooled sample and each subgroup. Odds ratios (with 95% CIs) for achieving a ≥ 1.3-point increase in LCQ total score are reported across subgroups.

## Results

### Baseline Characteristics and Demographics

Of 1360 participants, 678 were randomized to placebo and 682 were randomized to gefapixant 45 mg. Mean cough duration was ~ 11 years, 75% were female, 62% had a primary diagnosis of RCC, 71% had a cough severity ≥ 60 mm on the 100 mm cough severity VAS, and 51% had a baseline cough frequency ≥ 20 coughs/h. Comorbidities reported with cough included asthma (*n* = 571, 42%), gastroesophageal reflux disease (*n* = 538, 40%), and upper-airway cough syndrome (*n* = 86, 6%). The most frequently prescribed medications were similar between the placebo and gefapixant groups and consistent with prior treatments of cough-associated conditions [i.e., obstructive airway disease (71%), acid reflux-related disorders (54%), and nasal preparations (54%)].

### Pooled Overall Data from COUGH-1 and COUGH-2

Primary trial results showed that treatment with gefapixant 45 mg, but not gefapixant 15 mg, resulted in significant reduction of 24-h cough frequency; therefore, only results from the gefapixant 45 mg and placebo cohorts were assessed.

Gefapixant 45 mg reduced 24-h cough frequency [(estimated relative reduction, 18.6% (95% CI 9.2–27.1%)] and awake cough frequency [estimated relative reduction, 17.4% (95% CI 7.5–26.2%)] at Week 12. At Week 24, gefapixant 45 mg was associated with a higher LCQ response rate (i.e., ≥ 1.3-point increase in LCQ total score) than placebo [73.5% vs 67.0%; estimated odds ratio, 1.37 (95% CI 1.06–1.77%)].

### Objective and Subjective Efficacy Across Subgroups

**Fig. 1:**
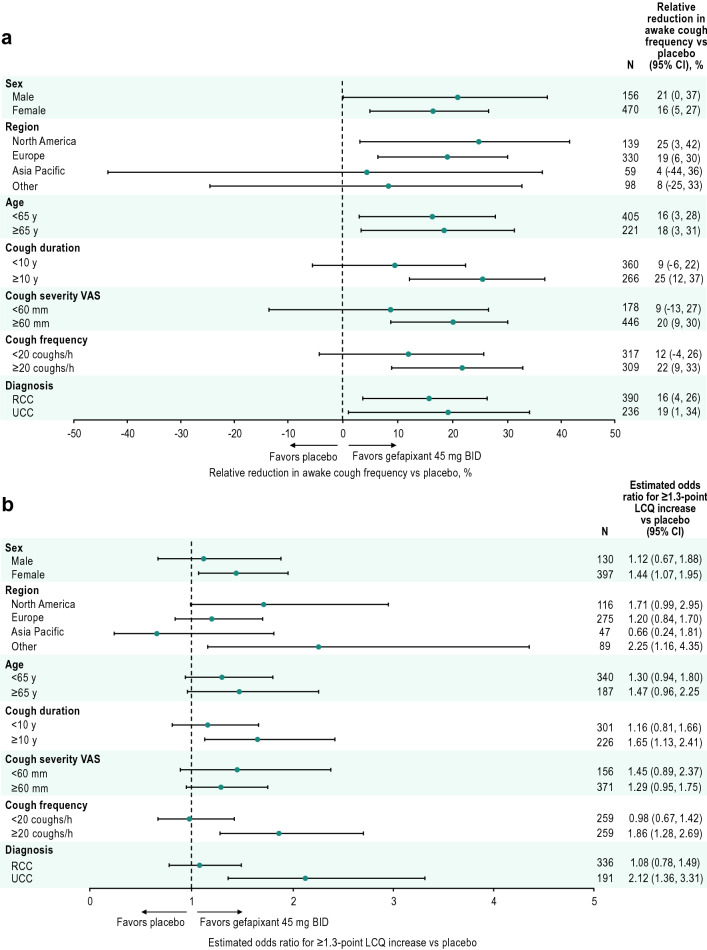
Objective and subjective efficacy in subgroups. **a** Relative reduction in awake cough frequency for gefapixant 45 mg BID vs placebo at Week 12 by subgroups and **b** odds ratios of achieving a ≥ 1.3-point increase from baseline in LCQ total score with gefapixant 45 mg BID vs placebo at Week 24 by subgroups. *BID* twice daily, *LCQ* Leicester Cough Questionnaire, *RCC* refractory chronic cough, *UCC* unexplained chronic cough, *VAS* visual analog scale

Across subgroups, those receiving gefapixant 45 mg demonstrated reductions in awake cough frequency at Week 12 relative to baseline [geometric mean ratios (Week 12/baseline) between 0.32 and 0.46, Table 1]. The range of responses among participants receiving placebo varied between geometric mean ratios of 0.39 and 0.54 across subgroups. Estimated relative reductions in awake cough frequency from baseline to Week 12 for gefapixant 45 mg relative to placebo are reported in Fig. 1a.

**Table 1:** Reduction in awake cough frequency at Week 12 by subgroup, reported as geometric mean ratio (Week 12/baseline)

Subgroup	Placebo		Gefapixant 45 mg BID		
	*n*	Geometric mean ratio (95% CI)	*n*	Geometric mean ratio (95% CI)	
Sex					
Male	161	0.46 (0.39, 0.55)	156	0.36 (0.31, 0.43)	
Female	480	0.48 (0.43, 0.52)	470	0.40 (0.36, 0.44)	
Region					
North America	148	0.53 (0.44, 0.64)	139	0.40 (0.33, 0.48)	
Europe	341	0.47 (0.42, 0.52)	330	0.38 (0.34, 0.43)	
Asia Pacific	58	0.42 (0.32, 0.56)	59	0.40 (0.30, 0.54)	
Other	94	0.39 (0.31, 0.49)	98	0.36 (0.29, 0.45)	
Age group					
< 65 years	415	0.44 (0.40, 0.49)	405	0.37 (0.33, 0.41)	
≥ 65 years	226	0.51 (0.45, 0.58)	221	0.41 (0.36, 0.47)	
Cough duration					
< 10 years	348	0.42 (0.37, 0.47)	360	0.38 (0.34, 0.42)	
≥ 10 years	293	0.54 (0.47, 0.61)	266	0.40 (0.35, 0.46)	
Cough severity VAS					
<60 mm	191	0.45 (0.39, 0.54)	178	0.42 (0.35, 0.49)	
≥60 mm	448	0.46 (0.42, 0.51)	446	0.37 (0.33, 0.41)	
Baseline 24-h coughs/h					
< 20 coughs/h	295	0.52 (0.46, 0.59)	317	0.46 (0.41, 0.52)	
≥ 20 coughs/h	346	0.41 (0.37, 0.46)	309	0.32 (0.29, 0.37)	
Diagnosis					
RCC	404	0.46 (0.42, 0.51)	390	0.39 (0.35, 0.43)	
UCC	237	0.48 (0.41, 0.56)	236	0.39 (0.33, 0.45)	

*BID* twice daily, *RCC* refractory chronic cough, *UCC* unexplained chronic cough, *VAS* visual analog scale

Across subgroups, ≥ 70% of participants treated with gefapixant 45 mg had a clinically meaningful increase in LCQ total score at Week 24 (range 70–81%, Table 2). The range of LCQ response rates for the placebo group varied between 60% and 82%. Odds of achieving a ≥ 1.3-point increase from baseline in LCQ total score with gefapixant 45 mg relative to placebo are reported in Fig. 1b.

**Table 2:** Percentage of participants defined as LCQ responders (≥ 1.3-point increase in LCQ score) at Week 24 by subgroup

	Placebo		Gefapixant 45 mg BID	
	*n*	Responders by subgroup, %	*n*	Responders by subgroup, %
Sex				
Male	134	69	130	72
Female	414	67	397	75
Region				
North America	124	61	116	74
Europe	286	70	275	73
Asia Pacific	50	82	47	75
Other	88	60	89	78
Age group				
< 65 years	351	69	340	76
≥ 65 years	197	64	187	72
Cough duration				
<10 years	304	72	301	76
≥10 years	244	62	226	72
Cough severity VAS				
< 60 mm	152	64	156	74
≥ 60 mm	394	69	371	74
Baseline 24-h coughs/h				
< 20 coughs/h	251	70	259	70
≥ 20 coughs/h	283	65	259	78
Diagnosis				
RCC	350	69	336	71
UCC	198	64	191	81

*BID* twice daily, *LCQ* leicester cough questionnaire, *RCC* refractory chronic cough *UCC* unexplained chronic cough, *VAS* visual analog scale

## Discussion

Previous predefined analyses were conducted to assess the primary endpoint of COUGH-1 and COUGH-2 (i.e., 24-h cough frequency) in protocol-defined subgroups [10]. The purpose of this analysis of additional endpoints (e.g., awake cough frequency, LCQ) in the same predefined subgroups was to determine if any one subgroup was driving overall trial results for these endpoints. Overall results demonstrate that gefapixant 45 mg improved 24-h cough frequency, awake cough frequency, and cough-specific QOL vs placebo in participants with RCC or UCC and, consistent with previous subgroup analyses reported for 24-h cough frequency [10], gefapixant 45 mg improved awake cough frequency and LCQ across predefined subgroups. Collectively, these results support the broad clinical benefit of gefapixant 45 mg in RCC and UCC. The main limitations were that sample sizes were not sufficiently powered to formally assess differences between subgroups with prespecified hypotheses, and sample sizes were limited for some subgroups or imbalanced between categories within subgroups (e.g., males vs females). Although statistical precision increases in a larger, pooled data set, apparent differences in efficacy between subgroups should be carefully interpreted for the reasons explained below.

Improvement with gefapixant 45 mg from baseline was generally consistent across subgroups. However, estimated relative reductions are impacted by both the treatment effect in the active group as well as the placebo response, which must be considered for interpretation. For example, although participants with a ≥ 10- vs < 10-year cough duration at baseline who were treated with gefapixant 45 mg had similar reductions in awake cough frequency from baseline (60–62%), participants with a baseline cough duration < 10 years experienced a greater placebo effect (58% reduction from baseline to Week 12) compared with those with a baseline cough duration ≥ 10 years (46% reduction), which led to a higher estimated relative reduction in awake cough frequency among participants with longer baseline cough duration. Both active treatment and placebo effects may have played a role in comparisons of estimated relative reductions in awake cough frequency between baseline cough frequency subgroups [i.e., those with < 20 coughs/h had a more moderate reduction from baseline than those with ≥ 20 coughs/h (54% vs 68% reductions) but also had a smaller placebo response (48% vs 59% reductions)]. The relative differences between these subgroups would therefore have been even more marked had the placebo responses been more comparable.

As a single-item assessment, the cough severity VAS is a relevant cough measure for clinical practice and was recently validated as a reliable and responsive measure in patients with chronic cough [12]. In the cough severity VAS subgroups, placebo responses were comparable (55% and 54% for < 60 and ≥ 60 mm, respectively), and those with more severe cough demonstrated a greater response to gefapixant compared with those with less severe cough; however, the numbers of participants in the subgroups were not well balanced and the confidence intervals were wide. Ultimately, all prespecified subgroups receiving gefapixant 45 mg experienced a ≥ 50% reduction from baseline in awake cough frequency, supporting a general improvement in cough regardless of baseline characteristics (Table 1).

Similar to awake cough frequency, improvements in LCQ with gefapixant 45 mg were generally consistent across subgroups, including LCQ response rates between 70% and 81% (Table 2). Just as those with higher cough frequency at baseline (≥ 20 coughs/h) had greater reductions in awake cough frequency compared with those with lower cough frequency (< 20 coughs/h), there were greater proportions of LCQ responders in those with higher vs lower baseline cough frequency. Additionally, in some subgroups, there were apparent differences between objective efficacy (i.e., awake cough frequency) and subjective efficacy (i.e., LCQ). The following considerations may explain this pattern of results. First, awake cough frequency and LCQ in this analysis were studied at different time points (i.e., Week 12 vs Week 24, respectively). Second, cough frequency is measured over 24-h, compared with a 2-week recall period for the LCQ, so these two endpoints may differ in sensitivity to change. Third, each outcome measures a different construct, and it is therefore not expected that the changes in LCQ and awake cough frequency would strongly correlate [13, 14]. Because no single measure can capture the full dimensions of the impact of chronic cough on patients, the use of both objective and subjective endpoints can provide richer detail regarding cough improvement and its effect on patients’ subjective experiences [15].

To conclude, data collected in the largest sample of individuals with RCC or UCC to date support the objective and subjective efficacy of gefapixant 45 mg, with relatively consistent improvements across sociodemographic and clinical-feature subgroups. Gefapixant, which is currently only approved in Japan for the treatment of RCC or UCC, may address a large unmet need.

## Data Availability

The data sharing policy, including restrictions, of Merck Sharp & Dohme LLC, a subsidiary of Merck & Co., Inc., Rahway, NJ, USA is available at http://engagezone.msd.com/ds_documentation.php. Requests for access to the clinical study data can be submitted through the Engage Zone site or via email to dataaccess@merck.com.
